# Ocular radiation exposure is negligible in normal volume endourological practice

**DOI:** 10.1308/rcsann.2024.0004

**Published:** 2024-03-06

**Authors:** J Peacock, J Henderson

**Affiliations:** Cheltenham General Hospital, UK

**Keywords:** Urolithiasis, Imaging, Percutaneous nephrolithotomy, Renal stone, Ureteral stones, Ureteroscopy

## Abstract

**Introduction:**

The annual dose limit for radiation exposure to the eye has been reduced recently; the eye is widely recognised as one of the most radiosensitive tissues in the body. There is minimal good quality research as to the radiation dose that the eye receives during endourological surgery and this study aimed to address this.

**Methods:**

A prospective study was performed over an 8-month period at a single large teaching hospital in the UK. Three index procedures were included: ureteric stent insertion, ureteroscopy (URS) and percutaneous nephrolithotomy (PCNL). Surgeons wore a dosimeter on the glabella with fluoroscopy time (FT) and dose area product (DAP) recorded for each case.

**Results:**

A total of 404 procedures were included (247 URSs, 150 ureteric stent insertions and 7 PCNLs). Dosimeters were worn by ten surgeons. Mean FTs (URS 20.56s; ureteric stent 18.96s; PCNL 360.67s) and mean DAP (URS 100.82cGy/m^2^, ureteric stent 119.82cGy/m^2^ and PCNL 1121.62cGy/m^2^) were identified with significant intersurgeon variability. No surgeon had a total dosimeter dose >0.00mSv.

**Conclusions:**

The International Commission on Radiological Protection recently reduced the yearly eye dose limit from 150 to 20mSv. Cataractogenesis is no longer considered a typical deterministic effect, with a threshold level below which no effect occurs. Even in higher volume centres, these annual limits are unlikely to be reached. Lead glasses may be considered for surgeons and radiologists with the highest exposure but, for the majority, ocular radiation exposure is negligible.

## Introduction

Endourology routinely utilises fluoroscopic guidance for diagnostic and therapeutic work. Radiation protection techniques are often inferred from other specialties, such as interventional radiology and cardiology. The overriding principle has always been to keep radiation exposure as low as reasonably achievable (ALARA). Despite this, a study of 165 urology trainees in the US revealed that only 53% felt adequately trained in radiation safety, with less than half (46%) correctly identifying the maximum acceptable physical exposure, and 70% of respondents had never worn a dosimeter. Although the thyroid and reproductive organs were more likely to be actively protected in the form of thyroid shields (73%) and body shields (99%), the eyes were often neglected when considering radiation safety, with almost no-one wearing lead-lined protective glasses.^1^ Until now, only one study has reported the mean lens dose measured over a year in urologists wearing regular dosimeters.^2^ This is despite there being widespread recognition of other risks to the eyes, such as splash and laser injury, with routine use of laser goggles being mandatory in all UK trusts.

The most significant risk of radiation exposure to the eye is radiation-induced cataract. Radiation-induced cataracts have long been recognised since the early days of radiation use. In Japanese atomic bomb survivors, cataract studies preceded cancer studies by several years. The lens of the eye is a unique organ, a nonvascular structure that does not lose any cells over its lifetime. There is no mechanism for the removal of damaged cells.^3^ The lens is considered to be the organ most sensitive to x-ray radiation.^4^ A cataract is defined as an opacity in the clear lens inside the eye that reduces the amount of incoming light. Cataracts result in a deterioration of vision, especially during daytime – often described as looking through a waterfall or waxed paper.

Although day-surgical replacement with an artificial lens can treat the condition, cataracts remain the dominant cause of blindness worldwide, with a considerable socioeconomic burden.^5^ Cataracts are classified anatomically into nuclear, cortical or posterior subcapsular (PSC) types. Age-related cataracts are typically of the nuclear or cortical subtype. Ionising radiation commonly produces PSC, and is more recently acknowledged as causing cortical cataracts.^6^

The International Commission on Radiological Protection (ICRP) has considered for over 60 years that the lens of the eye is among the most radiosensitive tissues, and has recommended dose limits for the lens to prevent occurrence of vision-impairing cataracts (VICs).^6^ In 1954, the ICRP recommended the first set of lens dose limits for workers and public and an effective depth of 3mm for the lens. In 1977, the ICRP classified cataracts as nonstochastic effects, later renamed ‘deterministic effects’ and then ‘tissue reactions’, with a threshold below which no effect would occur. Lens dose limits therefore aim to prevent VICs, but not minor opacities.

Occupational and public lens dose limits, respectively, have so far undergone eight and six revisions since 1954. The ICRP most recently updated their guidelines in 2011, after a number of studies on radiation caractogenesis revealed that cataracts may occur at much lower doses than previously thought. The threshold dose for radiation-induced eye cataracts was reduced to 0.5Gy for both acute and fractionated exposures, and a 7.5-fold reduction recommended in the dose limit for the eye lens for workers from 150mSv (millisieverts) to 20mSv per year, averaged over defined periods of 5 years, with no single year exceeding 50mSv.

## Aim

The aim of this study was to evaluate ocular radiation exposure in real-world practice. It is the responsibility of the employer to take all steps that are necessary to restrict employees’ exposure to ionising radiation and to ensure that doses are monitored. With such a dramatic reduction in threshold dose (0.5Gy) and recommended yearly dose limit for the eye (20mSV) in the most recent ICRP guidelines, the aim was to determine whether there is a need for protective ocular equipment in the form of mandatory lead-lined glasses.

## Methods

A prospective study was performed over an eight-month period in a large teaching hospital in the UK (Cheltenham General Hospital). Three urological fluoroscopy guided interventions were analysed: rigid cystoscopy and insertion of JJ stent, semi-rigid±flexible ureteroscopy (URS) (RURS/FURS) and percutaneous nephrolithotomy (PCNL). URS followed by ureteric stent insertion was recorded as URS.

**Figure 1:**
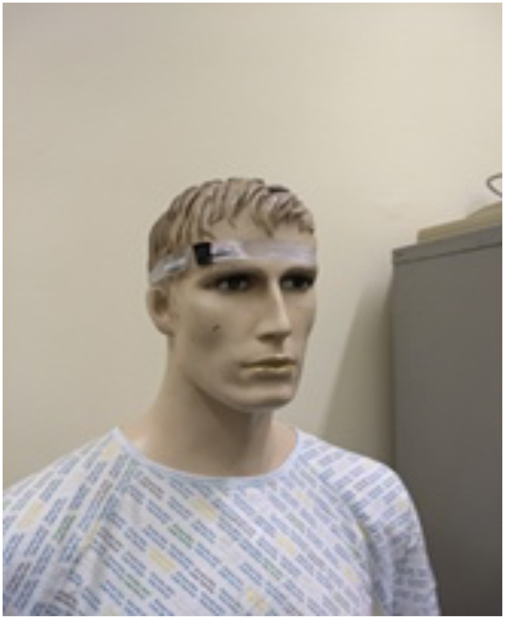
Dosimeter placed on forehead with TLD positioned on glabella TLD = the rmoluminescent dosemeter.

**Figure 2:**
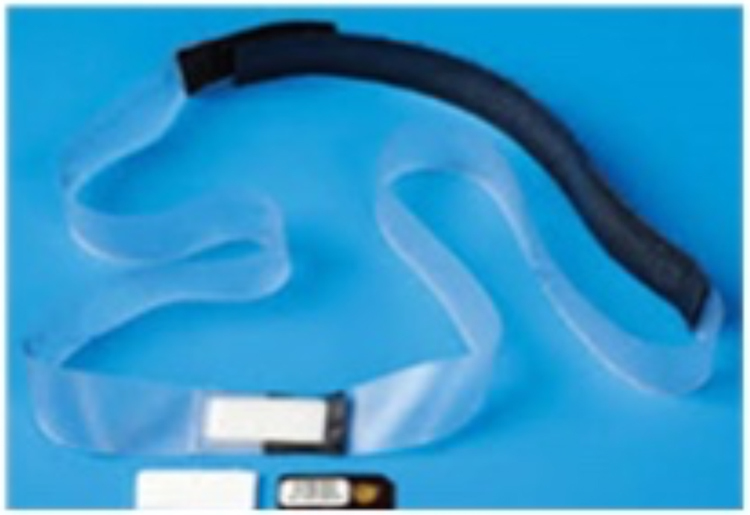
TLD in impermeable plastic headb and TLD = thermolu m inescent dose meter.

All urologists involved in a case wore personal dosimeters on the glabella as shown in Figure 1. Each surgeon was provided with their own, numerically labelled dosimeter. The dosimeters were supplied by Radiation Protection Services (RRPPS) of University Hospitals Birmingham NHS Trust. The dosimeters were thermoluminescent chips, enclosed in impermeable plastic headbands (Figure 2). If more than one urologist was present in the room, the role of each surgeon was recorded (primary surgeon, assisting surgeon). Once a case was completed, the surgeons were to immediately remove the dosimeter and store it in a safe location away from any form of x-ray radiation. Dosimeters were replaced on a two-monthly basis and returned to RRPPS for analysis. Results were made available on the online RRPPS approved dosimetry service.

All surgeons wore lead aprons and thyroid shields in line with trust policy. Surgeons recorded their training grade. Basic demographics and operative time were recorded for all patients with laterality if appropriate. Radiographers were made aware of the study and the need to record fluoroscopy time (FT, seconds) and dose area product (DAP, cGy/m^2^) for each case and to make the report available on the CRIS® Radiology Information System.

Patients for all rigid cystoscopy and URS cases were placed in the lithotomy position. All PCNL cases were carried out with the patient in the prone position. A mobile C-arm fluoroscopy unit was used in each case*.* A Ziehm vision flat panel detector image intensifier was positioned over the patient in all cases, with the x-ray tube under the table creating an ‘under the couch’ C-arm unit. Pulsed fluoroscopy mode, as opposed to constant fluoroscopy, was used in each case.

## Results

Data collection took place between 26 February 2020 and 1 October 2020. During this time period, 246 URS, 150 ureteric stent insertions and 7 PCNL took place, with data captured for all cases.

Of the URSs, 134 (54.3%) were combined RURS/FURS, 108 (43.7%) were RURS only and 4 (1.6%) cases FURS only (Table 1). The average age of all patients undergoing URS was 62.1 years. A registrar was the primary surgeon in 109 (44.1%) cases, with a consultant being the primary surgeon in the remaining 138 (55.9%). An assisting surgeon was present in 91 (36.8%) cases. Mean FT for all URS cases was 20.56s. Mean DAP was 100.82cGy/m^2^. For combined RURS/FURS cases, mean FT was 20.69s, mean DAP was 101.41cGy/m^2^_._ For FURS cases alone, mean FT was 138.48s, mean DAP was 262.37cGy/m^2^. For RURS cases alone, mean FT was 14.51s, mean DAP was 93.37cGy/m^2^.

**Table 1:** Mean FT and mean DAP for each operation

Operation	Mean age (years)	Mean FT (s)	Mean DAP (cGy/m^2^)
Combination RURS/FURS (*n*=134)		20.69	101.41
FURS alone (*n*=4)		138.48	262.37
RURS alone (*n*=108)		14.51	93.37
All URS (*n*=246)	62.1	20.56	100.82
Ureteric stent insertion (*n*=150)	66.7	18.96	119.82
PCNL (*n*=7)	53.14	360.67	1121.62

DAP = dose area product; FT = fluoroscopy time; FURS = flexible ureteroscopy; PCNL = percutaneous nephrolithotomy; RURS = semi-rigid ureteroscopy; URS = ureteroscopy.

For ureteric stent insertion, the average age of all patients was 66.7 years. A registrar was the primary surgeon in 91 (60.67%) of cases, with a consultant being the primary surgeon in the remaining 59 (39.33%). An assisting surgeon was present in 32 (21.3%) cases. Mean FT for all ureteric stent cases was 18.96s. Mean DAP (cGy/m^2^) was 119.82cGy/m^2^.

For PCNL, the average age of all patients was 53.14 years. A consultant was the primary surgeon in seven (100%) of cases. An assisting surgeon was present in two (28.5%) cases. Mean FT for all PCNL cases was 360.67s. Mean DAP was 1,121.62cGy/m^2^.

Dosimeters were worn by a total of ten surgeons during the study period: four consultants and six registrars. All four consultants have a subspecialty interest in Endourology. All four consultants surgeons wore their dosimeters for the entire study period. Three dosimeters were misplaced during the months of August and September among the registrar contingent; however, no significant data were lost during this period due to the inactivity of the surgeons.

**Figure 3:**
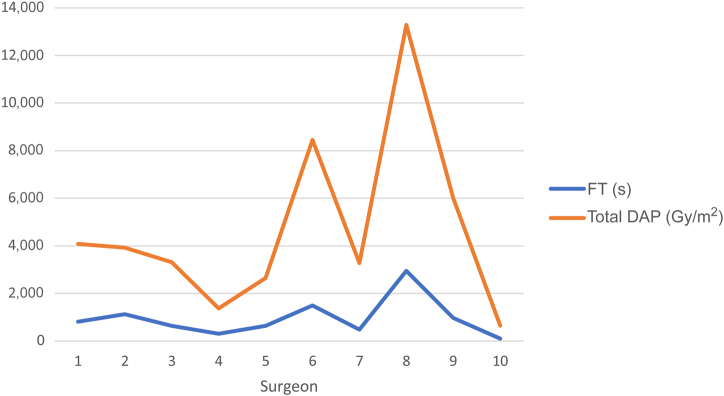
FT and total DAP per surgeon DAP = dose area product; FT = fluoroscopy time.

The number of cases for each surgeon is outlined in Table 2, along with total dosimeter recorded dose in mSv. The total number of procedures ranged from 6 to 68 (mean 39.5). During the study period, no surgeon exceeded 0.00mSv total dosimeter recorded dose. Individual FTs and DAP results for each surgeon are outlined in Table 2. The lowest total FT for all procedures was 100s (Surgeon 10), the highest time being 2,949s (Surgeon 8). These times were reflected in the corresponding total DAP results of 644.96cGy/m^2^ (Surgeon 10) and 13,283.13cGy/m^2^ (Surgeon 8) (Figure 3). As no surgeon exceeded 0.00mSv total dose, no surgeon reached the yearly limit of 20mSv recommended in the most recent ICRP guidelines.

**Table 2:** Summary of results: number of procedures, FT, DAP and recorded dose

Primary operating surgeon	RURS/FURS	RURS	FURS	Stent	PCNL	Total number of procedures	FT URS (s)	FT stent (s)	FT PCNL (s)	Total FT	DAP URS (cGy/m^2)^	DAP stent (cGy/m^2)^	DAP PCNL (cGy/m^2)^	Total DAP (cGy/m^2^)	Total recorded dose (mSv)
1	16	14	0	14	0	**44**	381	429	0	**810**	1,751.60	2,330.47	0	**4,082.07**	0.00
2	21	13	2	11	1	**48**	893	74	163	**1,130**	3,020.26	706.80	189.78	**3,916.84**	0.00
3	4	9	0	17	0	**30**	326	317	0	**643**	1,348.13	1,962.26	0	**3,310.39**	0.00
4	18	9	0	5	0	**32**	246	61	0	**307**	1,217.49	146.81	0	**1,364.30**	0.00
5	14	8	0	9	0	**31**	512	128	0	**640**	2,007.48	633.25	0	**2,640.73**	0.00
6	32	14	1	21	0	**68**	1,082	414	0	**1,496**	5,170.21	3,273.34	0	**8,443.55**	0.00
7	5	7	0	10	0	**22**	132	354	0	**486**	606.33	2,667.86	0	**3,274.19**	0.00
8	27	10	1	10	7	**55**	785	163	2,001	**2,949**	5,955.70	787.48	6,539.95	**13,283.13**	0.00
9	6	15	0	38	0	**59**	359	610	0	**969**	2,369.59	3,641.34	0	**6,010.93**	0.00
10	0	0	0	6	0	**6**	0	100	0	**100**	0	644.86	0	**644.96**	0.00

DAP = dose area product; FT = fluoroscopy time; FURS = flexible ureteroscopy; PCNL = percutaneous nephrolithotomy; RURS = semi-rigid ureteroscopy; URS = ureteroscopy.

## Discussion

In medicine, the number of interventional and surgical procedures requiring fluoroscopic guidance is rising. Medical fields with a risk of high radiation exposure such as interventional radiology and interventional cardiology have already taken measures to improve radiation safety after initial data demonstrated high doses. In a 15-year follow-up study, Spanish cardiologists noted a 14-fold decrease in radiation exposure with institution of a radiation protection programme and updated equipment with use of ceiling suspended films for further protection.^4^ Although lead aprons and thyroid shields are well adopted, an appreciation of the risks to Urologist's eyes is less well recognised—among European urology residents, only 3.2% reported wearing leaded eye protection on a regular basis.^7^

The lens is considered the organ most sensitive to x-ray radiation.^4^ Both posterior subcapsular and cortical cataracts are associated with ionising radiation exposure. Cataractogenic radiation damage occurs at the ‘germinative zones’ at the anterior surface where dividing cells form a clear crystalline-protein fibre that migrates towards the posterior pole of the lens—the PSC region.^6^

The aim of this study was to evaluate ocular radiation exposure in real-world conditions for the Endourologist. Until now, only one study has reported the mean lens dose measured in a year in urologists wearing regular dosimeters.^2^ In the eight-month period during which this study was carried out, no surgeon's dosimeter detected any significant radiation exposure, with no value >0.00mSv. All dosimeters were worn on the glabella of the surgeon, between the eyes, therefore it is reasonable to suggest that no significant radiation affected this region.

Simson *et al* defined recommended national reference levels for intraoperative radiation during stent insertion, ureteroscopy and PCNL. The FLASH study comprised 3,651 patients over 12 hospitals. Over a 12-month period, the highest volume centre performed 489 URSs and 73 ureteric stents; therefore, the 247 URSs and 150 ureteric stents included in this study over an eight-month period should enable reasonable comparison.

The DAP results in this study were below the median value for URS and ureteric stent in the FLASH study: URS 1.0Gy/cm^2^ vs 2.2Gy/cm^2^, ureteric stent 1.2Gy/cm^2^ vs 1.7Gy/cm^2^. For PCNL, the DAP was above the median value reported in the FLASH study (11.2Gy/cm^2^ vs 8.5Gy/cm^2^), although still below the recommended reference level for PCNL as suggested by FLASH (24.1Gy/cm^2^). The surgeon who performed the vast majority of the PCNLs and had the highest exposure to radiation (Table 2; surgeon 8) still had an <0.00mSv total recorded dose. This may offer some reassurance to other endourologists who perform more PCNLs per year than surgeon 8.

In this study, for all three procedures, the total FT was below the suggested national reference levels as per FLASH: URS 20.56s vs 57s, ureteric stent 18.96s vs 49s and PCNL 360.67s vs 431s. This demonstrates the judicious use of intraoperative radiation at this institution to ensure patient exposure is ALARA. Although promising, the dosimeter results in this study must be interpreted with caution. Not only are there higher volume centres in the UK, where the number of procedures and yearly radiation exposure will be greater, there is also great interhospital and intersurgeon variability of FT and DAP. There may be up to a 20-fold difference in median DAP between high and low-volume centres for PCNL.^8^ Furthermore, there are a number of patient factors, not evaluated in this study, that may affect radiation dose to both patient and surgeon; for example, it is recognised that severely obese patients receive threefold higher dose rates compared with nonobese patients.^9^

Induction of cataracts in humans has long been viewed as a deterministic effect that had a dose threshold and for which the severity increased and the latency decreased as the radiation dose increased above that threshold.^3^ Taylor *et al* reported that the threshold for a risk of cataract formation was 2,500mSv (eye lens cumulative equivalent dose) or 2.5Gy. These authors reported that it would take 50 years of normal urological surgery to reach that threshold and questioned the utility of lead glasses.^10^

However, the threshold for cataract formation has been repeatedly updated and lowered, with new insights into the early disease manifestations. The most recent guidelines state that the threshold dose for radiation-induced cataracts is 500mSv (0.5Gy) for both acute and fractionated exposures. A reduction was also recommended for the occupational annual equivalent dose from 150mSv to 20mSv, averaged over defined periods of 5 years (with no single year exceeding 50mSv).^11^ The threshold for acute exposure was judged as 0.5Gy mainly from two papers on the prevalence of cataracts and cataract surgery both at 55–57 years after exposure in A-bomb survivors. A threshold for highly fractionated or protracted exposure was judged also to be 0.5Gy mainly from one group, on cataracts 12–14 years after exposure in Chernobyl cleanup workers. However, the initial papers did not report on minor opacities; further analysis of the Chernobyl database revealed dose-threshold estimates (Gy) as low as 0.35Gy for posterior subcapsular stage 1 and 0.34Gy for superficial cortical stage 1 cataracts.^12^ Moreover, further analysis of A-bomb data suggested risks for cataracts necessitating lens surgery at doses far lower than 1Gy.^3^

Vano *et al* demonstrated posterior subcapsular lens changes characteristic of ionising radiation exposure in 50% of interventional cardiologists and 41% of nurses with a mean cumulative eye dose of 100mSv–18,900mSv, once again suggesting that the threshold may be even lower than expected. They concluded most lens injuries result after several years of work without eye protection.^13^ These findings are supported by a further large study including a 20-year follow-up of more than 35,000 radiological technologists who were assessed in terms of risk for eye lens opacification and cataract. The study provided evidence that exposure to relatively low doses of ionising radiation (lifetime dose up to 0.06Gy) may be harmful to the eye lens and increase the long-term risk of cataract formation. The findings suggested that the likelihood of cataract formation increases with increasing exposure to ionising radiation with no apparent threshold level.^11^

An important consideration is whether radiation-induced cataract really is a tissue reaction (deterministic effect), or whether it is in fact a stochastic one, or both. This further highlights the importance of Endourologists limiting their ocular radiation exposure. Operator age, years of practice and exposure, and lifetime dose may be additive risks for development of radiation-induced lens opacities. A more recent development is the suggestion of a genetic predisposition to radiation-induced cataract, with some being more susceptible than others.^14^

## Conclusion

In conclusion, this study demonstrated none of the surgeons achieving values near to the recommended ICRP guideline of 20mSv per annum. Even in higher volume centres, these annual limits are unlikely to be reached, with ocular radiation exposure negligible. Nevertheless, cataractogenesis is no longer considered a typical deterministic effect, with a threshold level below which no effect occurs, and it is therefore reasonable to suggest lead glasses be considered only for surgeons and radiologists with the highest exposure.
